# Molecular Epidemiology of Herpangina Children in Tongzhou District, Beijing, China, During 2019-2020

**DOI:** 10.3389/fmed.2022.822796

**Published:** 2022-04-25

**Authors:** Ming-Zhu Xie, Lin-Yi Chen, Yan-Na Yang, Yan Cui, Si-Hui Zhang, Tian-Shuo Zhao, Wan-Xue Zhang, Juan Du, Fu-Qiang Cui, Qing-Bin Lu

**Affiliations:** ^1^Department of Laboratorial Science and Technology and Vaccine Research Center, School of Public Health, Peking University, Beijing, China; ^2^Institute for Infectious Diseases and Endemic Diseases Prevention and Control, Beijing Tongzhou Center for Disease Control and Prevention, Beijing, China

**Keywords:** molecular epidemiology, herpangina, children, China, COVID-19 pandemic

## Abstract

**Background:**

The changing pattern of pathogen spectrum causing herpangina in the time of coronavirus disease 2019 (COVID-19) pandemic was unknown. The purpose of this study was to investigate the changes on the molecular epidemiology of herpangina children during 2019-2020 in Tongzhou district, Beijing, China.

**Method:**

From January 2019 to December 2020, children diagnosed with herpangina were recruited by the staff from Tongzhou Center for Disease Control and Prevention (CDC) in Beijing. Viral RNA extraction from pharyngeal swabs was used for enterovirus (EV) detection and the complete VP1 gene was sequenced. The phylogenetic analysis was performed based on all VP1 sequences for EV genotypes.

**Result:**

A total of 1,331 herpangina children were identified during 2019-2020 with 1,121 in 2019 and 210 in 2020, respectively. The predominant epidemic peak of herpangina children was in summer and autumn of 2019, but not observed in 2020. Compared to the number of herpangina children reported in 2019, it decreased sharply in 2020. Among 129 samples tested in 2019, 61 (47.3%) children were detected with EV, while 22.5% (20/89) were positive in 2020. The positive rate for EV increased since June 2019, peaked at August 2019, and decreased continuously until February 2020. No cases were observed from February to July in 2020, and the positive rate of EV rebounded to previous level since August 2020. Four genotypes, including coxsackievirus A6 (CV-A6, 9.3%), CV-A4 (7.8%), CV-A10 (2.3%) and CV-A16 (10.1%), were identified in 2019, and only three genotypes, including CV-A6 (9.0%), CV-A10 (6.7%) and CV-A16 (1.1%), were identified in 2020. The phylogenetic analysis showed that all CV-A6 strains from Tongzhou located in Group C, and the predominant strains mainly located in C2-C4 subgroups during 2016-2018 and changed into C1 subgroup during 2018-2020. CV-A16 strains mainly located in Group B, which consisting of strains widely distributed around the world.

**Conclusions:**

The predominant genotypes gradually shifted from CV-A16, CV-A4 and CV-A6 in 2019 to CV-A6 in 2020 under COVID-19 pandemic. Genotype-based surveillance will provide robust evidence and facilitate the development of public health measures.

## Introduction

Herpangina is an acute infectious disease in children characterized by acute fever and herpes ulcers in the pharynx. It is a self-limited disease, but also causes severe symptoms and death (1–3). Herpangina mainly appears in children under 5 years old with a 5–10 days course of illness. It occurs frequently in summer. Herpangina is most prevalent in Asian countries, such as China, Thailand and Japan (4). The main pathogen causing herpangina is enteroviruses (EV) and the predominant genotypes include coxsackievirus A2 (CV-A2), CV-A4, CV-A5, CV-A6, CV-A8, CV-A10, CV-A16 and enterovirus A71 (EV-A71). CV-B1, CV-B2, CV-B3, CV-B4 and CV-B5 were also reported in a few cases (2). Pooling the studies conducted in Asia and France, the meta-analysis showed that the detection rates of CV-A2, CV-A6, CV-A4, CV-A10, CV-A16, and EV-A71 in the children with herpangina were 31.3, 17.6, 17.4, 15.8, 11.7, and 5.6% during 2005–2019, respectively (4).

Herpangina has not been included in the China National Notifiable Infectious Disease Reporting System, and it is not a priority disease to control in China (4). In 2018, we found that the predominant pathogen of herpangina was CV-A6, followed by CV-A10 and CV-A4 in Beijing, indicating that the pathogen spectrum of herpangina was changing to CV-A6 (5). During the pandemic of coronavirus disease 2019 (COVID-19), many countries have implemented multiple non-pharmaceutical interventions to control pandemic, such as masks wearing, hands washing, tele-working and so on (6). The comprehensive measures and people's caution could reduce the spread of respiratory infectious diseases (7). During the COVID-19 pandemic, the positive detection rate of influenza decreased significantly in China, the United States, Australia, Chile and South Africa (8). The incidence of hand, foot and mouth disease (HFMD), chickenpox, mumps and other infectious diseases also decreased rapidly in Jiangsu province, China in 2020 compared with that during 2017–2019 (9). However, the changing pattern of pathogen spectrum causing herpangina under the COVID-19 pandemic remains unknown.

Therefore, we attempted to illuminate the changes of the molecular epidemiology in children with herpangina by using the 2019–2020 herpangina surveillance data in Tongzhou district, Beijing.

## Materials and Methods

### Patients

This study was performed using the surveillance data from Tongzhou Center for Disease Control and Prevention (CDC) in Beijing. The children under 14 years diagnosed with herpangina by clinician were recruited from January 2019 to December 2020. The inclusion criteria of participants were that children with typical symptoms of herpangina, including sore throat, fever, herpes or ulcers on the palatal arch, soft palate, uvula and tonsil, but not on the hand, foot or trunk (2). The samples were randomly collected for the detection from about 10% herpangina children reported in the surveillance by the trained professionals and there were at least five samples per month. The children without the pharyngeal swabs were excluded. The CDC staff collected the basic demographic data and laboratory test results of these children by the uniform questionnaire, including gender, birthdate, onset date, sampling date, and diagnosis.

This study was approval by Peking University Institutional Review Board Office (the number IRB00001052-19005). Oral informed consents were obtained from children's guardians before recruitment in this study.

### Viral RNA Extraction and Detection

The children's pharyngeal swabs were collected within 5 days of onset for nucleic acid extraction. The QIAamp MiniElute Virus Spin Kit (Cat. No.: 57704, QIAGEN, Hilden, Germany) was used to extract RNA following the instructions of the manufacturer. The RNA was stored in a 1.5 mL RNase-Free fresh EP tube at −80°C.

We amplified RNA by a set of broad-spectrum primers for the 5′ untranslated region (5′UTR) of EVs (5). The detection of EV and further classification of EV-A71 and CV-A16 for EV-positive samples were performed by real-time polymerase chain reaction (PCR), respectively. Then, specific primers based on VP1 gene were used for the amplification of specimens positive for CV-A4, CV-A6, CV-A10 and CV-A16 by nested reverse transcription PCR. All primers and reaction conditions selected in the experiment were designed according to the previous report (5). The amplicons products were visible on gels by electrophoresis, and positive products were sent for bidirectional sequencing using Sanger method (5).

### Sequence Analysis

Lasergene's DNA SeqMan software (version 7.1.0, DNA Star Inc. Madison, WI, USA) was used to assemble the identified nucleotides sequences. VP1 sequences of CV-A4, CV-A6, CV-A10 and CV-A16 in GenBank were downloaded. All sequences identified in this study were submitted to the GenBank database (OL470931-OL470940 for CV-A4, OL470904-OL470913 for CV-A6, OL470914-OL470921 for CV-A10 and OL470922-OL470930 for CV-A16). BioEdit 7.0 and MEGA (Version 7.0.14) were used to align and compare sequences, delete strain types with high homology while maintained regional diversity (10). Phylogenetic tree was constructed using maximum likelihood method with 1000 bootstrap replications and the distance between different branches were calculated.

### Data Analysis

Non-normal continuous variables were described by median and interquartile range (IQR). Categorical variables were described by frequency and proportion. One-way analysis of variance, Chi square test, Fisher's exact test or non-parametric test was used for statistical comparison between groups. Stata 17.0 (Stata Corp LP, College Station, TX) was used for all statistical analyses. A two-sided *P* < 0.05 was statistically significant.

## Results

### Demographics Characteristics

**Figure 1:**
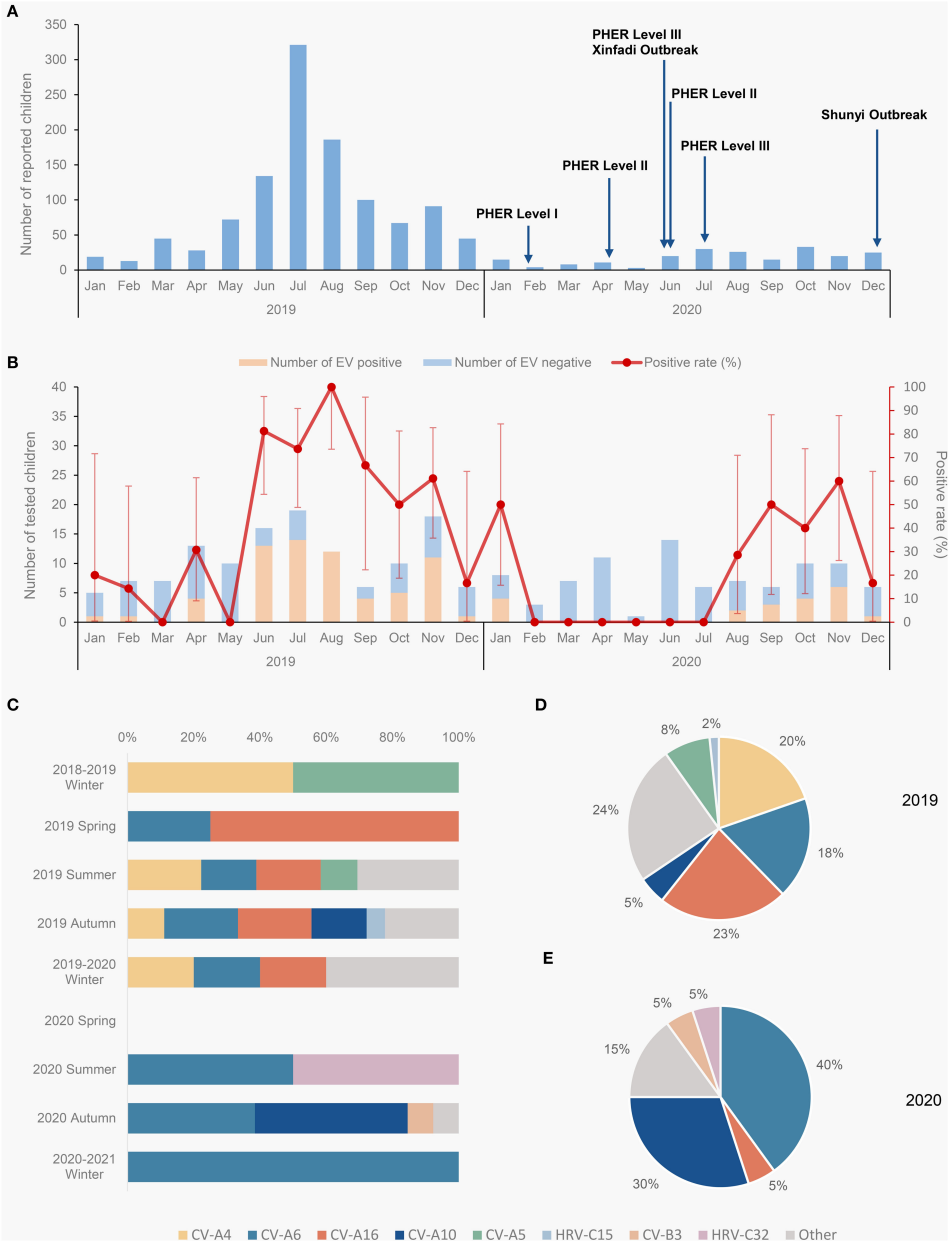
Molecular epidemiology of herpangina children in Tongzhou during 2019-2020. **(A)** Monthly reported herpangina children in 2019 and 2020; **(B)** monthly detection of herpangina in 2019 and 2020; **(C)** composition of enterovirus types by season 2019-2020; **(D)** composition of enterovirus types in 2019; **(E)** composition of enterovirus types in 2020. The 2018-2019 winter includes January and February 2019, 2019-2020 winter includes December 2019, January and February 2020, the 2020-2021 winter includes December 2020. PHER, Public health emergency response.

Totally 1,331 herpangina children were identified in Tongzhou district, Beijing during 2019-2020, including 1,121 in 2019 and 210 in 2020 (Figure 1A). The epidemic peak of herpangina children occurred in summer and autumn of 2019, which was not observed in 2020. The number of herpangina children decreased sharply in 2020 that was smaller all the year compared to that in 2019.

Among reported cases, 218 herpangina samples were collected for molecular detection, including 129 in 2019 and 89 in 2020. Among the collected samples, the median age of children was 46 (IQR 27–60) months and 46.5% (60/69) were boys in 2019, while the median age was 48 (IQR 2–72) months and 44.9% (40/49) were boys in 2020 (Table 1). No significant differences were observed on the clinical manifestations between the 2 years.

**Table 1:** The demographical characteristics and clinical manifestations of herpangina children in 2019 and 2020.

**Characteristics**	**Total (*N* = 218)**	**2019 (*n* = 129)**	**2020 (*n* = 89)**	***P*-value**
Age, months, median (IQR)	46 (24–64)	46 (28–60)	48 (20–72)	0.524
0–36	80 (36.7)	43 (33.3)	37 (41.6)	0.573
36–60	70 (32.1)	54 (41.9)	16 (18.0)	
≥60	68 (31.2)	32 (24.8)	36 (40.5)	
Gender, boy, *n* (%)	100 (45.9)	60 (46.5)	40 (44.9)	0.820

### Enterovirus Genotypes

The detection rate of EV among the herpangina children was 37.16% (81/218) during 2019-2020, with 47.3% (61/129) in 2019 and 22.5% (20/89) in 2020 (*P* < 0.001). The detection rate for EV increased since June 2019, peaked at August 2019, and then decreased until February 2020 (Figure 1B). And it maintained at zero from February to July in 2020, then rebounded to previous level since August 2020.

The herpangina pathogens were further tested among the samples. CV-A6, CV-A16, CV-A4, CV-10, CV-A5, CV-B3, human rhinovirus C15 (HRV-C15) and HRV-C32 were detected while all samples were negative for EV-A71. These four genotypes, including CV-A6 (19), CV-A16 (15), CV-A4 (12) and CV-10 (9), accounted for the largest proportion. Other types of pathogens included CV-A5 (5), CV-B3 (1), HRV-C15 (1) and HRV-C32 (1). In 2019, there were 61 samples detected positive, including CV-A4 (12), CV-A16 (14), CV-A6 (11), CV-A10 (3), CV-A5 (5), HRV-C15 (1) and other types (15). In 2020, there were 20 samples detected positive, including CV-A16 (1), CV-A6 (8), CV-A10 (6), CV-B3 (1), HRV-C32 (1) and other types (3).

Among the cases in 2019, four genotypes were identified with the positive rate of 9.3% for CV-A6 (12/129), 7.8% for CV-A4 (10/129), 2.3% for CV-A10 (3/129) and 10.1% for CV-A16 (13/129). While in 2020, three genotypes were identified as follows: CV-A6 (8/89, 9.0%), CV-A10 (6/89, 6.7%) and CV-A16 (1/89, 1.1%). No co-infections were observed during 2019-2020.

In 2019, the predominant genotypes were CV-A16 (23.0%), CV-A4 (19.7%) and CV-A6 (18.0%), observed in summer, autumn and winter (Figures 1C,D). In 2020, CV-A6 (accounted for 40.0%) became the predominant genotype since summer 2020, and CV-A10 was observed accounting for a large proportion in autumn (Figures 1C,E).

### Phylogenetic Analysis

**Figure 2:**
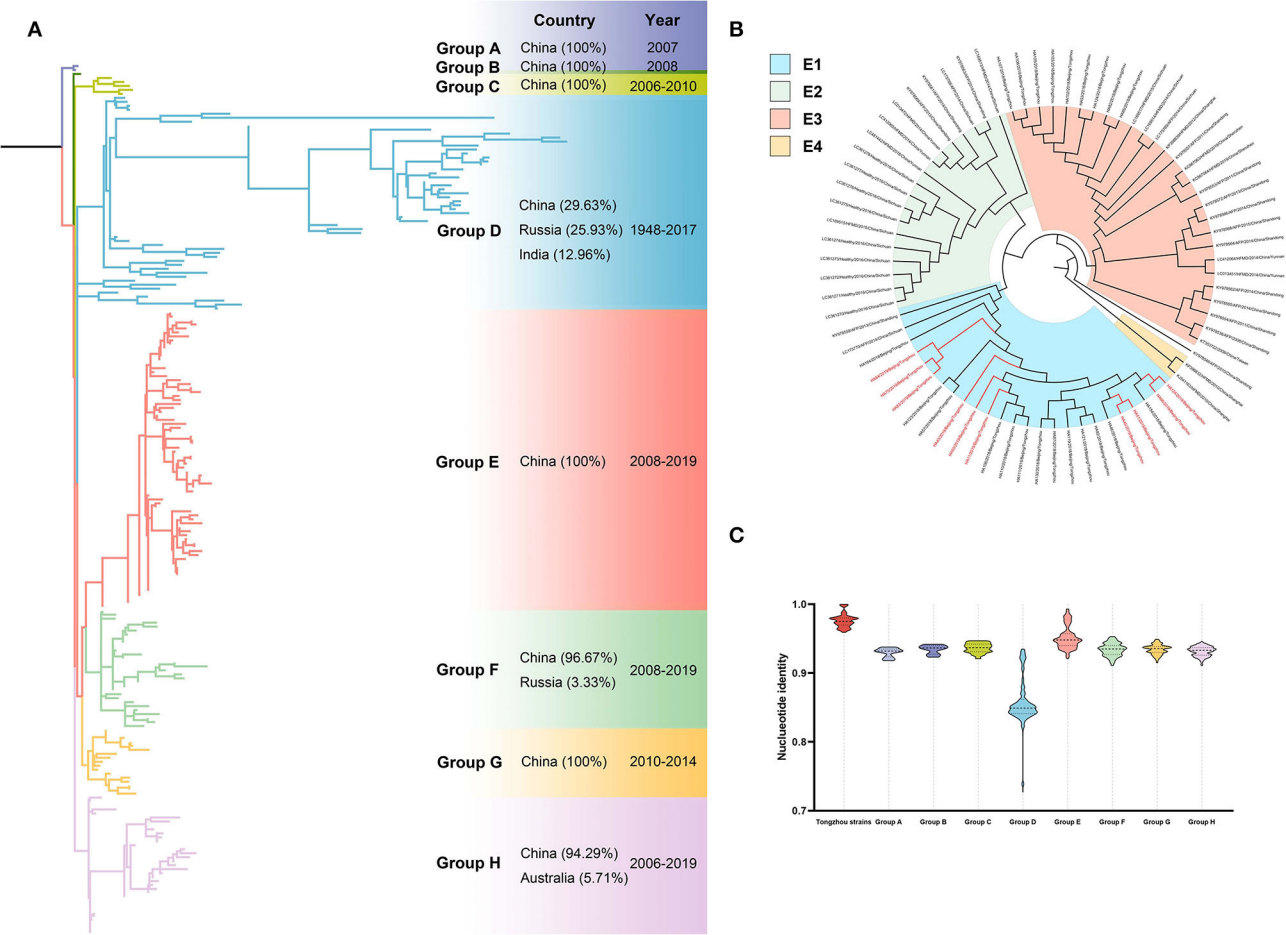
Phylogenetic tree of CV-A4 based on VP1 gene. Different colors represented different groups, and the red branches represented amplified strains from this study. We used maximum likelihood estimation with 1000 bootstrap replications to construct phylogenetic trees. **(A)** All CV-A4 strains were divided into eight groups; countries and times of the strains with the highest frequency in each group were listed on the right. **(B)** Group E of the CV-A4 phylogenetic tree. **(C)** Nucleotide identities between Tongzhou amplified CV-A4 strains and different groups. Each violin graph showed the nucleotide identities between intragroup of amplified strains and different groups based on VP1. Solid line represents median, while the dashed line represents the quartiles.

Totally ten CV-A4 positive samples were amplified for phylogenetic analysis with 209 VP1 sequences downloaded from GenBank. Phylogenetic tree showed eight groups (A-H) and more than 90% sequences were from China except in Group D (29.63%, 16/54) (Figure 2A). All sequences from Tongzhou located in Group E composed with strains completely from China, which was further divided into four subgroups (E1~E4) (Figure 2B). All strains from Tongzhou in this study located in subgroup E1 composed of strains mainly from Beijing during 2018-2019 and two strains from Shandong and Sichuan province. Spatiotemporal characteristics revealed that the predominant strains mainly located in E2-E4 subgroups between 2008 and 2017 and shifted into E1 subgroup during 2018-2019. The sequences were high homological within the group, as well as groups from A to H except Group D exceeded 90% (Figure 2C).

**Figure 3:**
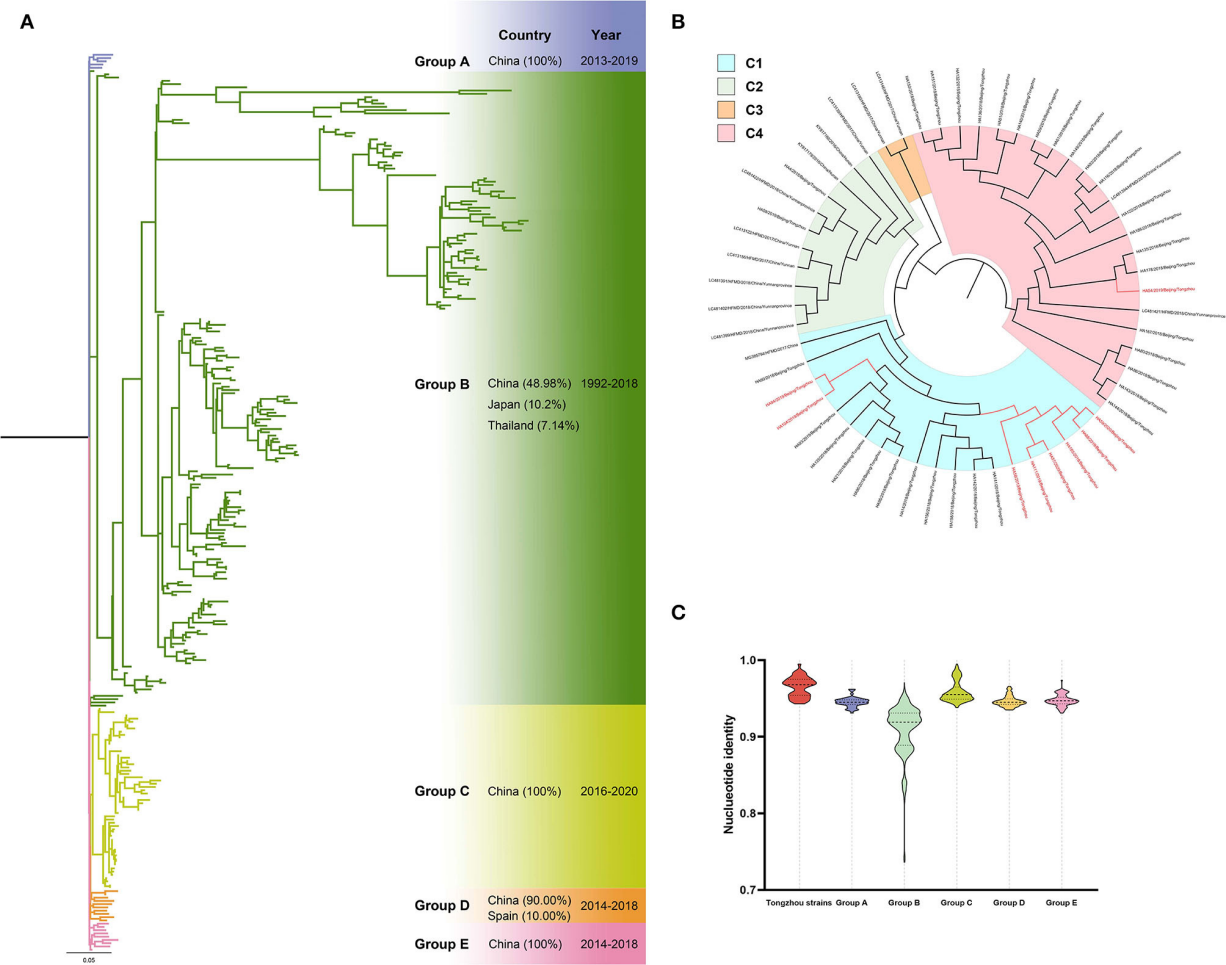
Phylogenetic tree of CV-A6 based on VP1 gene. Different colors represented different groups, and the red branches represented amplified strains from this study. We used maximum likelihood estimation with 1000 bootstrap replications to construct phylogenetic trees. **(A)** All CV-A6 strains were divided into five groups; countries and times of the strains with the highest frequency in each group were listed on the right. **(B)** Group D of the CV-A6 phylogenetic tree. **(C)** Nucleotide identities between Tongzhou amplified CV-A6 strains and different groups. Each violin graph showed the nucleotide identities between intragroup of amplified strains and different groups based on VP1. Solid line represents median, while the dashed line represents the quartiles.

Ten CV-A6 positive samples (eight in 2019 and two in 2020) were amplified for phylogenetic analysis with 266 VP1 sequences from GenBank. Phylogenetic tree showed five groups (A-E) and more than 90% sequences of these groups were from China except in Group A (48.47%, 95/196) (Figure 3A). All the ten sequences in this study located in Group C including nine in subgroup C1 and one in subgroup C4 (Figure 3B). Spatiotemporal analyses revealed that, the predominant strains mainly located in C2-C4 subgroups during 2016-2018, while transferred into C1 subgroup during 2018-2020. The sequences were high homological within the group, and groups from B to D exceeded 93% (Figure 3C).

**Figure 4:**
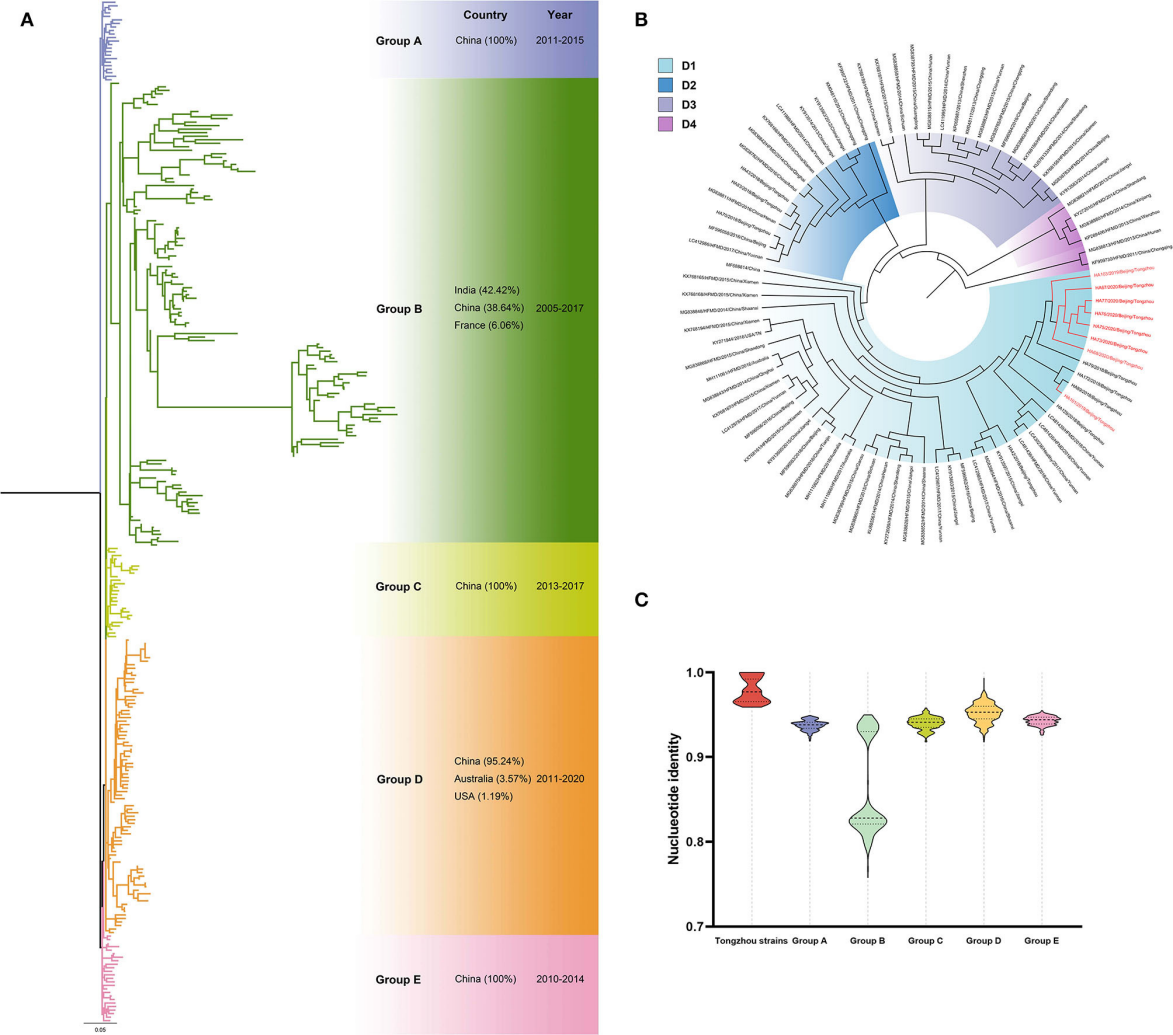
Phylogenetic tree of CV-A10 based on VP1 gene. Different colors represented different groups, and the red branches represented amplified strains from this study. We used maximum likelihood estimation with 1000 bootstrap replications to construct phylogenetic trees. **(A)** All CV-A10 strains were divided into five groups; countries and times of the strains with the highest frequency in each group were listed on the right. **(B)** Group D of the CV-A10 phylogenetic tree. **(C)** Nucleotide identities between Tongzhou amplified CV-A10 strains and different groups. Each violin graph showed the nucleotide identities between intragroup of amplified strains and different groups based on VP1. Solid line represents median, while the dashed line represents the quartiles.

Eight CV-A10 positive samples (two in 2019 and six in 2020) were amplified for phylogenetic analysis with 282 sequences from GenBank. Phylogenetic tree showed five groups (A-E) and more than 95% sequences in these groups were from China, except those in Group B (38.64%, 51/132) (Figure 4A). All sequences from Tongzhou located in Group D, and specifically in subgroup D1 (Figure 4B). The strains in subgroup D1 were mainly from China and a few from Australia and the USA. Furthermore, all the strains in this study located in subgroup D1a. Spatiotemporal analyses revealed that epidemic strains mainly located in D2-D4 subgroup during 2011-2013, while shifted into subgroup D1 during 2014-2020. The sequences were high homological within the group, and groups from A to E except Group B exceeded 91% (Figure 4C).

**Figure 5:**
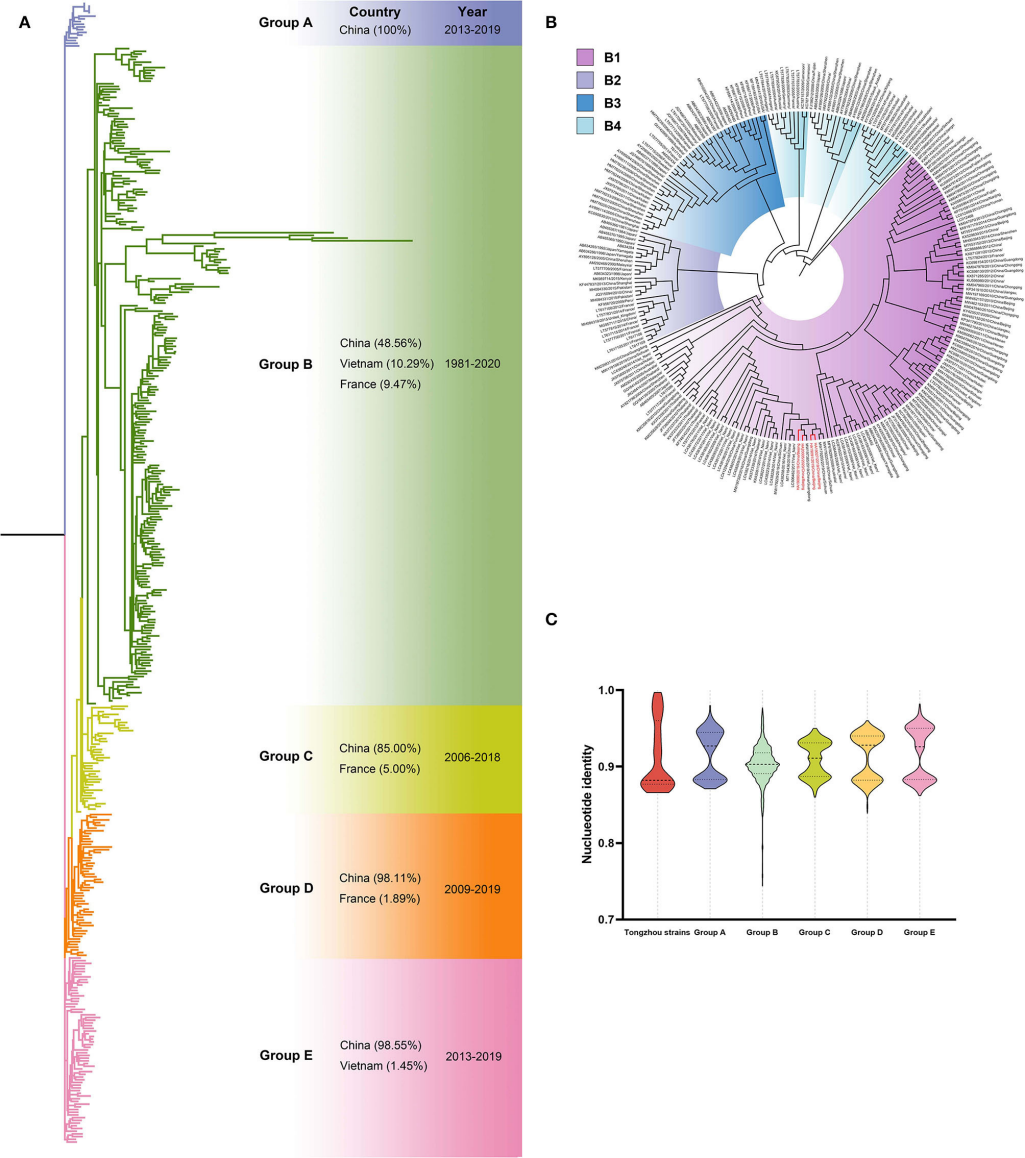
Phylogenetic tree of CV-A16 based on VP1 gene. Different colors represented different groups, and the red branches represented amplified strains from this study. We used maximum likelihood estimation with 1000 bootstrap replications to construct phylogenetic trees. **(A)** All CV-A16 strains were divided into five groups; countries and times of the strains with the highest frequency in each group were listed on the right. **(B)** Group B of the CV-A16 phylogenetic tree. **(C)** Nucleotide identities between Tongzhou amplified CV-A16 strains and different groups. Each violin graph showed the nucleotide identities between intragroup of amplified strains and different groups based on VP1. Solid line represents median, while the dashed line represents the quartiles.

Nine CV-A16 positive samples (eight in 2019 and one in 2020) were amplified for phylogenetic analysis with 413 VP1 sequences from GenBank. Phylogenetic tree showed five groups (A-E) (Figure 5A) and more than 98% sequences were from China except Group B (48.56%, 118/243) and Group C (85.00%, 34/40). The sequences from Tongzhou located in Group A, B, D and E, respectively. Four sequences located in Group B, including three from 2019 and one from 2020. Furthermore, the four sequences located in subgroup B4 (Figure 5B). The sequences had high homologies within the group, and groups from A to E except Group B exceeded 83% (Figure 5C).

Compared with sequences from Tongzhou in 2018, CV-A4 sequences in 2019 had over 92.1% homologies (Supplementary Figure 1A), CV-A6 and CV-A10 sequences had over 89.9% homologies, while CV-A16 sequences had over 87.9% homology in 2019 and 2020 (Supplementary Figure 1B). There were several amino acid mutations in four genotypes, as following: K18R and K274R from 2018 to 2019 for CV-A4; V29A from 2018 to 2020 for CV-A6; S14N, A23V, I283V and T284A from 2018 to 2020 for CV-A10; M23K, T164K, V251I and V284I from 2018 to 2020 for CV-A16.

## Discussion

A review showed that the herpangina outbreaks were reported in China, Thailand, Korea, Japan and France since 2009, and the pathogens varied from year to year and region to region (4). For example, CV-A2 caused herpangina outbreak in Hangzhou, 2010 (11), while CV-A6 caused outbreaks in Guangzhou and Shanghai in 2015-2017 (12). The outbreaks occurred in Beijing in 2014 and 2016 was mainly caused by CV-A4 (13), and CV-A10 (14).

In this study, no sample was EV-A71 positive while CV-A4, CV-A6, CV-A10 and CV-A16 were the four main enterovirus types. The pathogenic spectrum of herpangina has changed largely between 2019 and 2020, with the main pathogen shifted from CV-A4, CV-A6, CV-A16 to CV-A6. The proportions of CV-A4 and CV-A16 decreased while CV-A6 and CV-A10 increased from 2019 to 2020. These four genotypes were also with the highest frequency in France during 2014-2015 (15). CV-A6 was detected a high proportion in HFMD or herpangina children in vast locations and time span (16), and it was the predominant pathogen in Guangdong in 2015, Singapore in 2013-2018 and Guizhou in 2019 (17, 18). Studies (19) showed that CV-A6 detection rate was low in 2010-2012, then increased in 2013, 2015, 2017 and remained high in 2018, while decreased in 2014, 2016 and 2019. Combined with that study, our result of CV-A6 detection rate increasing from 2019 to 2020 reflected that CV-A6 strains rose epidemic peak every 2 years. The reason of this phenomenon was not clear yet but there were some possible explanations. One was because children obtained antibody after CV-A6 infection, so in the year after peak, the children were less susceptible. Researchers proposed that unique amino acid substitutions of CV-A6 strains might lead to changes in virological characteristics such as antigenicity and had been emerged every 2 years to cause epidemics (20). As there were no samples tested EV-A71 positive, our result did not conform with some findings, where EV-A71 was with a large proportion (21, 22). This may result from territorial differences, revealing the necessity of strengthening disease surveillance.

Pathogen changes among herpangina samples may be caused both by the COVID-19 pandemic and natural immunity. During the COVID-19 pandemic, strict prevention measures were adopted in China, including wearing masks, reducing public gathering, and having schools closed, etc. These measures also cut off the transmission chain of viruses causing herpangina, and in turn led to the lower number of children and the absence of seasonal epidemic peak in 2020. High prevalence of CV-A16 in 2019 may lead to a high immunity in population and a low prevalence of CV-A16 in the following year.

Compared to 2018, the positive rate for EV in 2019-2020 decreased, that indicated the preventive measures against herpangina were effective in China. The positive rate for EV decreased to zero in February 2020 and lasted for 5 months, synchronized with the implementation of COVID-19 pandemic intervention policy. When the policy was loosened on 20 July 2020 by adjusting the public health emerging response level II to level III (23), which allowed opening libraries, museums, gyms by constraining 50% visitors flow and opening exhibitions, sports events, the positive rate of EV increased to the same level in 2019. This suggests that it may worth adopting similar strategy in herpangina prevention during epidemic season.

This study showed that CV-A16 strains in Group B spread worldwide, including China (118), Vietnam (25), France (23) and other 20 countries. We identified four sequences located in subgroup B4, and the branch also contained strains from Vietnam and Thailand since earlier years, which might indicate these strains were imported to China from these two countries.

There was different amino mutation at site 29 (A29T) reported in CV-A6 VP1 in Xiamen (2009-2015) (24). Same mutations A23V and I283V of CV-A10 were found in Xiamen (2009-2014) (25). Mutation L23M was found in CV-A16 in China before 2018 (26), M23K detected in this study may be the further mutation. It is known that VP1 involved in receptor binding and antigenicity property. Those amino acid mutations on VP1 may affect the viral functions (25, 26). However, the specific function of those mutations remains unclear.

There were some limitations in this study. First, the number of amplification samples was small, which might bias the results. Second, we did not amplify the whole genome of each genotype that could reflect genome variations. Third, 81 children were positive for EV testing but 18 samples failed to genotyping. This may be due to low virus load or the limitation of amplification conditions. Therefore, the EV genotyping techniques should be improved, so as to contribute to the disease monitoring.

## Conclusion

Our study found that the incidence of herpangina in Tongzhou district, Beijing decreased in the time of COVID-19 pandemic, while the predominant strains of herpangina were CV-A6, CV-A16, CV-A4 and CV-A10 from 2019 to 2020. The genotypes shifted from CV-A16, CV-A4 and CV-A6 in 2019 to CV-A6 in 2020. Genotype-based surveillance will provide robust evidence and facilitate the development of public health measures. Vaccines against EVs, especially for coxsackieviruses, should be developed as soon as possible.

## Data Availability Statement

The datasets presented in this study can be found in online repositories. The names of the repository/repositories and accession number(s) can be found in the article/Supplementary Material.

## Ethics Statement

The Peking University Institutional Review Board Office granted Ethical approval to carry out the study within its facilities (IRB00001052-19005). Written informed consent from the participants' legal guardian/next of kin was not required to participate in this study in accordance with the national legislation and the institutional requirements.

## Author Contributions

M-ZX, L-YC, and Y-NY collected and analyzed the data, prepared figures and tables, authored drafts of the article, and approved the final draft. M-ZX, L-YC, Y-NY, S-HZ, T-SZ, and W-XZ performed the experiments. Q-BL, F-QC, and JD conceived and designed the experiments, reviewed drafts of the article, and approved the final draft. All authors have read and agreed to the published version of the manuscript.

## Funding

This work was supported by Fundamental Research Funds for the Central Universities and Peking University Health Science Center (BMU2021YJ041), Peking University Medicine Fund of Fostering Young Scholars' Scientific and Technological Innovation (BMU2021PY005), and Joint Research Fund for Beijing Natural Science Foundation and Haidian Original Innovation (L202007).

## Author Disclaimer

The views expressed in the article are those of the authors and do not necessarily reflect the position of the funding bodies.

## Conflict of Interest

The authors declare that the research was conducted in the absence of any commercial or financial relationships that could be construed as a potential conflict of interest.

## Publisher's Note

All claims expressed in this article are solely those of the authors and do not necessarily represent those of their affiliated organizations, or those of the publisher, the editors and the reviewers. Any product that may be evaluated in this article, or claim that may be made by its manufacturer, is not guaranteed or endorsed by the publisher.

## Supplementary Material

Supplementary Figure 1 Nucleotide identities of 2018 and 2019/2020 Tongzhou strains based on VP1 genes. Each violin graph showed the nucleotide identities between different genotypes Tongzhou strains in 2018 and 2019/2020 based on VP1. Solid line represented median, while the dashed line represented the quartiles. **(A)** Comparison of nucleotide homology of strains from Tongzhou in 2018 and 2019, for CV-A4, CV-A6, CV-A10 and CV-A16. **(B)** Comparison of nucleotide homology of strains from Tongzhou in 2018 and 2020, for CV-A6, CV-A10 and CV-A16.
