# Supplementary figures and images for: Molecular Epidemiology of Herpangina Children in Tongzhou District, Beijing, China, During 2019-2020
Source: Front Med (Lausanne). 2022 Apr 25;9:822796. doi: 10.3389/fmed.2022.822796 (PMC9082675; doi:10.3389/fmed.2022.822796)

**A**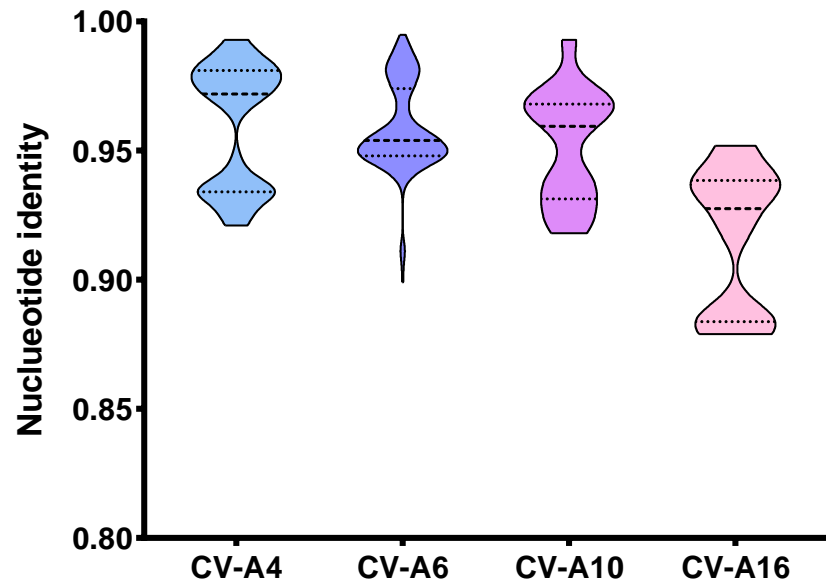**2019****B**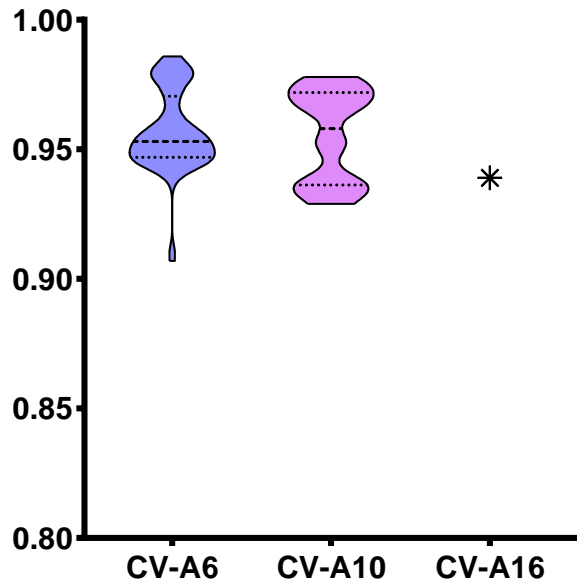**2020**

Supplement: Supplementary Figure 1 — Nucleotide identities of 2018 and 2019/2020 Tongzhou strains based on VP1 genes. Each violin graph showed the nucleotide identities between different genotypes Tongzhou strains in 2018 and 2019/2020 based on VP1. Solid line represented median, while the dashed line represented the quartiles. (A) Comparison of nucleotide homology of strains from Tongzhou in 2018 and 2019, for CV-A4, CV-A6, CV-A10 and CV-A16. (B) Comparison of nucleotide homology of strains from Tongzhou in 2018 and 2020, for CV-A6, CV-A10 and CV-A16. [file Image_1.PDF]
